# The Nursing Department in the Annual Report

**Published:** 1921-09-03

**Authors:** 


### THE MATRONS' AND SISTERS' DEPARTMENT.

### THE NURSING DEPARTMENT IN THE ANNUAL REPORT.

The claims of the nurse training-schools on public attention become more insistent every year. Financially the nursing department has assumed an importance which cannot be ignored. Enormous capital sums have in some cases to be raised to provide accommodation for the nursing staff. Many new nurses' homes are in contemplation; some are already completed, and few, indeed, are the institutions which have not been compelled recently to make expensive alterations. With all this it is not surprising that the nursing staff in modern and well-equipped hospitals should occupy the attention of subscribers. We welcome the marked change which is taking place in the preparation of annual reports in regard to the nursing staff. With a few conspicuous, and we must add deplorable, exceptions, the interest of governors and subscribers in the nurse training-schools they are expected so largely to subsidise is now recognised as reasonable, and efforts are made to show in what degree the necessary expenses incurred in the training of nurses are justified by results.

We have before us the matron's report of the Royal Liverpool Infirmary, which in many respects is a model well worthy of imitation. It gives all the information which subscribers are justified in expecting, yet in no way departs from the wise reticence which has become a tradition in the nursing services.

We note first that the number of entrants, thirty-four, is stated, with the number of nurses, twenty-four, who completed their training. We have a list of lecturers, and the prize-winners in each course, with information that the senior nurses received instruction and passed in sick-room cookery, and that special classes were held by the matron's assistant, the home sisters, and special sisters. We should welcome also the names of the examiners. We learn that the health of the nurses has been particularly good—a point of great interest, for the report circulates largely among prospective candidates—and that only thirty-seven, all minor ailments, passed through the doctor's hands, of whom but a few had to go to the sick-room. So much for the probationers.

The next point of interest for subscribers is the future of the women trained at considerable cost to be nurses. What, they are entitled to know, became of the twenty-four women who completed their course? Miss Cummins supplies the information, and very interesting it is. Four out of the number joined, or rather remained on, the nursing staff of the Royal Infirmary; three went on to take midwifery training; seven passed into the ranks of private nurses near home; four went to Edinburgh, one to take a sister's post; two returned home; one joined the Indian Q.A.I.M.N.S.; one the Ministry of Pensions Nursing Service; one took up a health appointment.

The next matter for sympathisers with the hospital to hear about is the year's progress from the point of view of nursing. Details tending to show the intimate connection of the training-school with medical and surgical progress help to increase the cordiality with which claims made by the nursing staff upon the finances will be met. We learn that at the Liverpool Royal Infirmary the work of the orthopædic department continues to increase, and that there are now twenty-two fully-certificated masseuses working. The temporary lapse of the School of Massage is explained as due to the present dearth of teachers consequent on the longer period of training required by the Chartered Society of Massage and Medical Gymnastics. We learn, too, that a third nurse has been added to the *x*-ray department, and that the staff now comprises a sister, two certificated nurses, and one in training. The number of nurses has been slightly increased to enable the fifty-six-hour week to work more smoothly.

The report does not neglect the consideration of the future. A sketch plan is given of the projected Nurses' Home, and an appeal is forecasted, with the committee's cogent reasons for not delaying the work.

Lastly, though by no means least in importance, we have the separate financial statement which enables the entire cost of the nursing department to be ascertained, though we must admit that we should prefer to find the cost of the private nurses' department separately entered. The committee, calling attention to the fact that out of the net increase of £1,427 in expenditure shown by the accounts nearly £900 is accounted for by the increase in salaries, confidently reckon on the public joining in their desire to be fair and just to those on whose efficiency and general contentment so much depends.

